# Genetic Insights Into Leukemia Susceptibility in the Arab Population: A Scoping Review

**DOI:** 10.7759/cureus.67421

**Published:** 2024-08-21

**Authors:** Abdulrahman Algarni

**Affiliations:** 1 Department of Medical Laboratory Technology, Faculty of Applied Medical Sciences, Northern Border University, Arar, SAU

**Keywords:** arab, disease genetics, middle eastern countries, genetic association, leukemia

## Abstract

As per the Global Cancer Observatory, the WHO Eastern Mediterranean region (which includes the Arabic countries) ranks highest for age-standardized mortality rate at 4 per 100,000, thus indicating a probable role of genetic associations. Identifying the genes associated with leukemia in the Arab population is crucial for effective preventive and treatment strategies. This scoping review aimed to determine the nature and extent of research available on the genes associated with the major types of leukemia among the Arab population. As per the scoping review guidelines, a comprehensive search was conducted in PUBMED and Google Scholar for articles published before 01/10/2023 and focused on leukemia-related genes among the Arab population. In total 119 studies, focusing on genes associated with leukemia met the inclusion criteria. On reviewing these studies, 27 genes were found to be associated with ALL, 33 genes with AML, seven genes with CLL, and 14 genes with CML. The majority of these genes were associated with an increased risk for the disease. Notably, the 119 studies covered only nine out of the 22 Arab countries, with 56 studies carried out in Egypt, exhibiting an imbalance in the regional distribution of the research landscape. Thus, indicating the inadequacy of research on leukemia genetics in the Arab region in comparison to the Western studies. This finding highlights the need for extensive research in the Middle Eastern region to gain geographically heterogeneous genetic information about the Arab population. In conclusion, this scoping study highlights the genes associated with the major types of leukemia among the Arab population and also indicates the need for comprehensive and regionally balanced research on leukemia genetics in Middle Eastern countries. Addressing this gap is essential to provide robust genetic data that can be used for targeted interventions to improve leukemia outcomes in the Middle East. Increased research efforts in all Middle Eastern countries will contribute to a greater understanding of genetic predisposition and help develop effective prevention strategies and treatments tailored to this population.

## Introduction and background

Leukemia is a term used for cancer related to the blood cells. It is a type of blood cancer, marked by altered hematopoietic progenitors and widespread infiltration of the bone marrow. Depending on which type of blood cell is affected, leukemia is further classified into lymphoid or myeloid. Furthermore, as per the rate of cell multiplication it is further classified as acute or chronic. Thus, the primary categories of leukemia comprise acute lymphoblastic leukemia (ALL), acute myeloid leukemia (AML), chronic lymphocytic leukemia (CLL), and chronic myeloid leukemia (CML) [1-3].

With a global incidence rate of 2.6 and 474,519 cases, leukemia ranks 13th globally among the types of cancers [4]. This incidence rate has shown regional variation, thus strengthening the significance of different environmental and genetic associations on the incidence of leukemia. According to data from the Global Cancer Observatory, in 2020, there were 311,594 deaths globally attributed to leukemia. Furthermore, the WHO Eastern Mediterranean region which also includes the Arab nations exhibited the highest age-standardized mortality rate, standing at four per 100,000 individuals. The age-standardized incidence rate was 5.2 per 100,000 for this region. The WHO Eastern Mediterranean region consists of the Middle Eastern or Arabic countries (except Iran, Pakistan, and Afghanistan). This region constitutes 22 countries and shows a clear difference in incidence rates and mortality rates of leukemia indicating an underlying genetic implication as per ethnicity [4-6].

Taking into account these ethnic disparities, this review aims to primarily assess the extent of research published on genes associated with leukemia relevant to the Arab population. The present review incorporates published data from 22 Arab countries (as enlisted in the Leagues of Arab States), including Algeria, Bahrain, Comoros, Djibouti, Egypt, Iraq, Jordan, Kuwait, Lebanon, Libya, Mauritania, Morocco, Oman, Palestine, Qatar, Saudi Arabia, Somalia, Sudan, Syria, Tunisia, the United Arab Emirates, and Yemen [7].

## Review

Search strategy and selection criteria

To understand the ethnic differences in leukemia observed in Middle Eastern countries, it is imperative to identify the genes demonstrating a strong association with leukemia. This scoping review aims to assess the extent, range, and nature of research published concerning leukemia genetics among the Arab population. The scoping review was carried out as per the guidelines of Preferred Reporting Items for Systematic Reviews and Meta-Analyses (PRISMA) - Scoping review (PRISMA-ScR) [8]. In this review, available literature was systematically scanned for studies examining leukemia patients in 22 countries, as mentioned earlier. All the searches were conducted between 1st - 31st October 2023 and included all studies published before 1st October 2023. The search was carried out in PubMed and Google Scholar using combined Medical Subject Headings (MeSH) indexing terms covering four main concepts such as “leukemia,” “genes,” “genetic association,” and “Arab” (and the name of the individual countries). Relevant synonyms for MeSH terms, indicated as Entry Terms on the MeSH page, were incorporated as text/word terms. In addition, search terms were applied to automatically filter out articles not published in English, studies exclusively focusing on animals (excluding human subjects), and article types that were deemed irrelevant due to lack of controls such as conference abstracts, comments, editorials, letters, newspaper articles, case reports, and similar materials.

Screening

Results were exported from PubMed and Google Scholar to Rayyan [9] and Excel (Microsoft) for screening and data extraction. Initially screening involved reviewing article titles and abstracts in Rayyan. For the article to be included, it required to provide data for at least one statistically significant genetic association with leukemia (with the mention of the type of leukemia). Articles were excluded from data extraction if they met any of the following criteria: lacked human data; reported only gene expression data; focused on karyotype or chromosome loci-related data; discussed outcomes related to treatment regimens; described genetic associations related to prognosis; were case reports, case series, or ecological studies; were letters, editorials, commentaries, or news articles; examined genetic associations in non-human subjects (animals, cell lines, or pathogens); demonstrated no statistically significant associations of relevance; were not available in full-text format; or were not available in English. Secondary data extraction from review articles was not carried out to avoid duplication. Following the screening of abstracts and titles, a thorough review was conducted for the resulting 119 articles.

Data extraction

Details included general study description (publication date and location of study), sample age and size, study design, type of leukemia, type of technique used, details of genes studied (gene name, rs ID, and genotype) measurement of the association (type of association) were extracted from the publication. Only statistically significant results were extracted; statistical significance was considered for *P* < 0.05.

Results

Studies Identified

**Figure 1:**
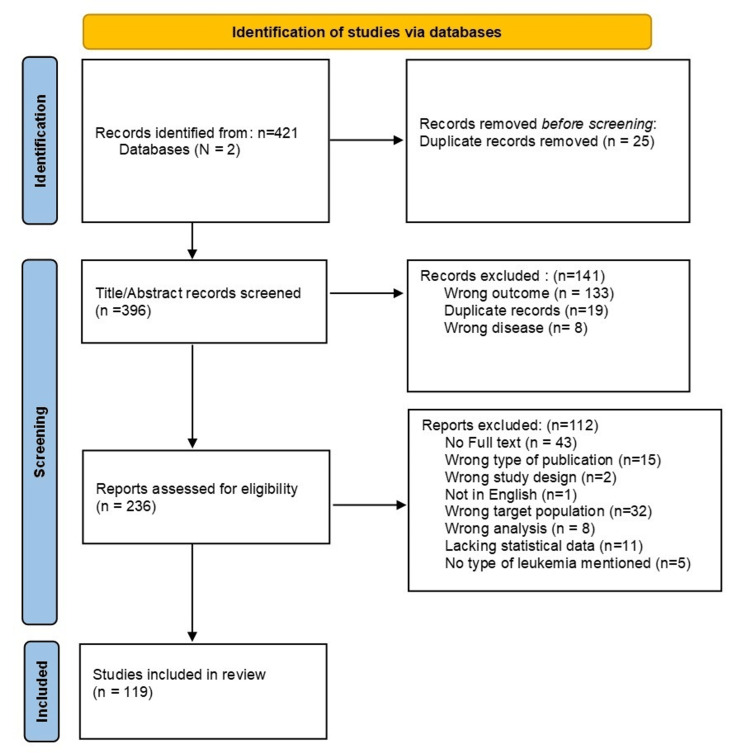
Flow diagram depicting the process of paper selection.

The initial stage involving the search in two different databases identified 421 articles. A total of 25 duplicate articles were excluded leaving a total of 396 articles. These 396 articles were screened by reviewing the title and abstract. Articles were excluded for the following reasons: treatment outcome focus (n = 64), outcomes not related to genetic associations (n = 39), gene expression studies (n = 17), karyotype-specific results rather than gene-specific (n = 9), focus on disease prognosis (n = 9), not focused on leukemia (n = 8), and duplicate records (n = 19). Upon further screening of the full text of the remaining 236 articles, exclusions were made for the following reasons: non-availability of the full article (n = 43); the article was a dissertation, case report, case series, communication, systematic review, or meta-analysis (n = 15); non-English language (n = 1); wrong target population (n = 32); analysis did not match the research question (n = 8); irrelevant statistical analysis (n = 11); and no mention of the type of leukemia (n = 5) (Figure 1). Thus, in total 119 articles were included for further analysis.

Distribution of the Studies Included for Analysis

**Figure 2:**
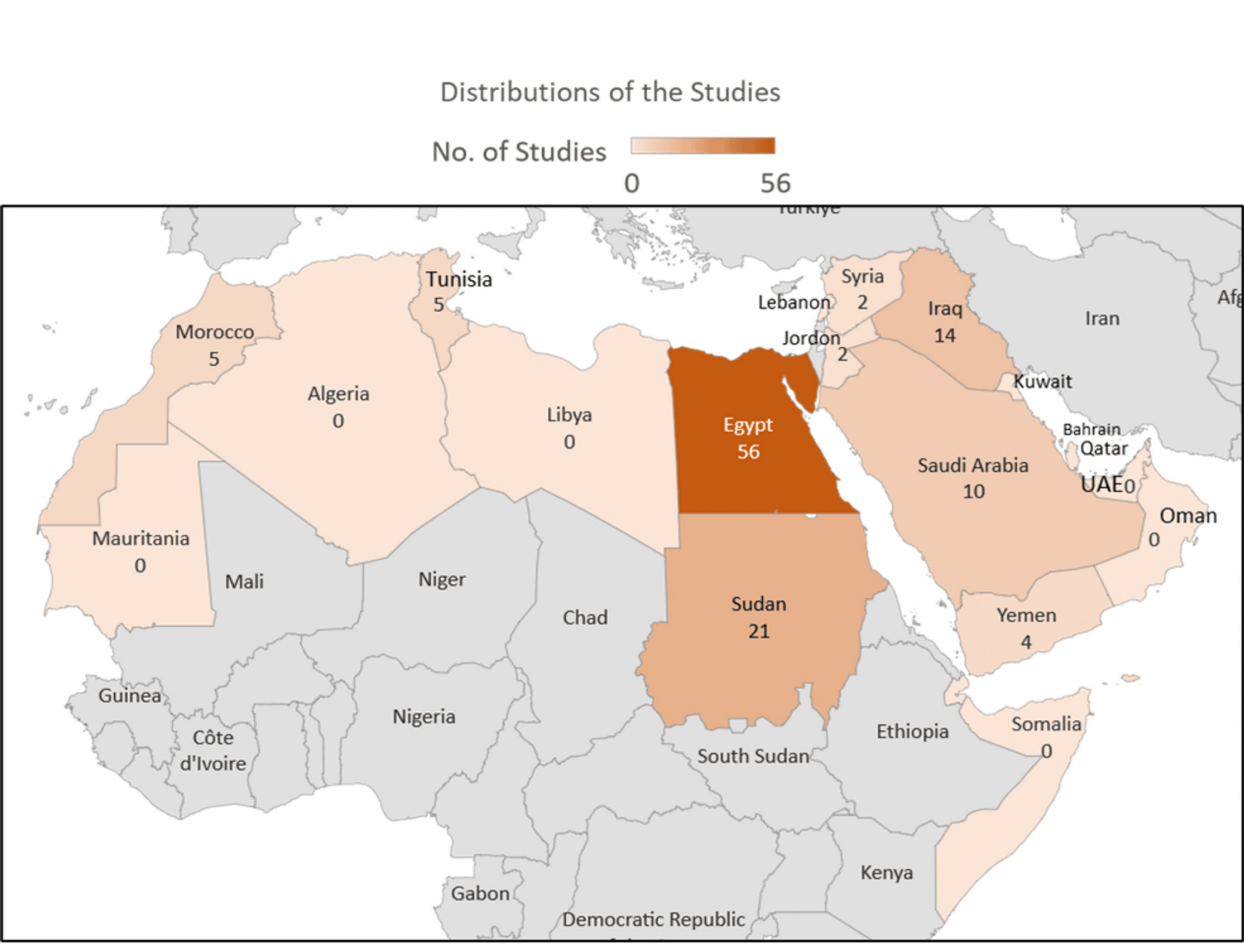
Distribution of the studies included for analysis. The figure was created using Microsoft Excel.

The region for this scoping review includes the Middle Eastern countries comprising 22 countries. The studies included in this review were from only nine countries in the Middle Eastern region (Figure 2). The majority of the studies were from Egypt (56/119) representing approximately 47% of the population being studied in the current review. Ten studies were conducted on the population of Sudan. No eligible study was found in Algeria, Bahrain, Comoros, Kuwait, Lebanon, Libya, Mauritania, Oman, Palestine, Qatar, Somalia, Sudan, and the United Arab Emirates, thus indicating a requirement for a study that is more representative of the Arab population.

Genes Associated With ALL Among the Arab Population

A total of 45 studies exhibiting genetic associations with ALL in the Arab population were identified (Table 1). These studies included 41 case-control studies of which 21 involved children (0-18 years), 12 examined adults, and eight studies investigated both adults and children (Appendix A). Additionally, four cross-sectional studies were identified, of which three examined children (<18 years) (Appendix A). On reviewing these articles, 27 genes were shown to be significantly associated with ALL. Of these 27 genes, variants of 25 genes were associated with increased risk for ALL, and variants of two genes appeared to reduce the risk for ALL (Table 2). Additionally, two alleles of HLA Class 1 appeared to be linked with a lower risk for ALL while the rest of its alleles seemed to increase the risk for ALL (Appendix A). Alleles of 19 of these genes were observed to increase the risk for childhood ALL (cALL). Eleven studies showed no significant associations with ALL with the genes studied and hence were not included in Table 2 [10-20].

**Table 1:** Distribution of the studies as per the type of leukemia. ALL, acute lymphoblastic leukemia; AML, acute myeloid leukemia; CLL, chronic lymphocytic leukemia; CML, chronic myeloid leukemia

Type of leukemia	No. of studies found	No. of genes associated
ALL	45	27
AML	45	33
CLL	11	7
CML	24	14

**Table 2:** Genes significantly associated with ALL. ALL, acute lymphoblastic leukemia

Sr. no.	Gene	Country	Type of association with ALL (+/-)	Reference
1	ARID5B	Egypt, Yemen	ALL (+)	[21,22], [23]
2	CCND1 (G870A)	Egypt	ALL (+)	[24]
3	CD19	Iraq	ALL (+)	[25]
4	CDKN2A	Yemen	ALL (+)	[26]
5	CYP1A1*4	Egypt	ALL (+)	[27]
6	Factor V Leiden	Egypt	ALL (+)	[28]
7	FOXP3	Egypt	ALL (+)	[29], [30]
8	GATA3	Egypt	ALL (+)	[31]
9	GSTM1	Sudan, Egypt, Yemen	ALL (+)	[32], [33], [34], [35]
10	GSTO2	Egypt	ALL (+)	[36]
11	GSTP1	Yemen	ALL (+)	[35]
12	GSTT1	Yemen, Sudan	ALL (+)	[32], [34], [35]
13	HFE H63D	Egypt	ALL (+)	[37]
14	HLA class I (A, B, Cw) and class II (DR, DQ) genes	Iraq	ALL (+)	[38], [39]
15	IKZF1	Yemen,Tunisia	ALL (+)	[26], [40]
16	IL-10	Sudan	ALL (+)	[41]
17	IL-15	Egypt	ALL (+)	[42]
18	IL-17A	Saudi Arabia	ALL (-)	[43]
19	KIR2DS4	Saudi Arabia	ALL (+)	[44]
20	MDR1	Egypt, Iraq,	ALL (+)	[33], [45], [46], [47]
21	MTHFR	Egypt	ALL (-)	[48], [49]
22	NAT2	Egypt	ALL (+)	[50]
23	NQO1	Tunisia, Saudi Arabia	ALL (+)	[51], [52]
24	Ras mutations	Iraq	ALL (+)	[53]
25	ROR-γ	Egypt	ALL (+)	[29]
26	TCF3-PBX1	Iraq	ALL (+)	[53]
27	TNFα	Sudan	ALL (+)	[54]

Four studies examined the association of GSTM1 with ALL and showed that the combination of GSTT1 null and GSTM1 null exhibited a 15-fold increase in risk among children (Appendix A) [32]. Additionally, the ARID5B (rs4948488) gene with genotype A/G + G/G showed the strongest association with a 22-fold increase in risk for developing ALL (Appendix A). The A/G genotype of ARID5B also seemed to increase the risk of developing ALL in adults by 21 times [23]. Along with the study carried out by Gamaleldin and Ibaby, two other studies also showed a significant association between the variant of the ARID5B gene and ALL [21-23]. Other genes showing significantly high risk in children were CCND1 (G870A) [24], MDR1 (C3435T) [46], CYP1A1*4 [27], and NQO1 (C465T) [52] (Table 1).

The variants of two genes showing protective association towards ALL were IL-17A (rs3748067) [43] and MTHFR C677T [48,49] (Appendix A). Two alleles of HLA class I (HLA-DQA 40101 allele and HLA-DQB130201 allele) also showed a protective association with ALL [39]. Understanding the role these genes play in reducing the risk for ALL would help in better prognosis and preventive measures.

Genes Associated With AML

In this review, 45 studies examining genes associated with AML in Middle Eastern countries were identified (Table 1). Of these 45, 44 were case-control studies, including 12 that focused on children (Appendix B). One cross-sectional study was identified which examined children in Iraq. From these studies, variants of 33 genes were found to be significantly associated with AML (Table 3). Variants of all these genes except for that of CYP1A1, increased the risk for AML. Two genetic variations among these genes viz. APE1 rs1130409T/T and ERCC2 (Lys751Gl) Allele T showed a protective association with AML. 13 studies on AML showed genes not to be associated with AML and hence were not included in Table 3 [16,19,55-65].

**Table 3:** Genes associated with AML in Arab countries. AML, acute myeloid leukemia

Sr. no.	Gene	Country	Type of association with AML (+/-)	Reference
1	APE1	Egypt, Iraq	AML (+)	[66], [67]
2	CASP9 712	Egypt	AML (+)	[68]
3	CYP1A1	Sudan	AML (-)	[69]
4	CYP2B6	Egypt	AML (+)	[70]
5	CYP3A4	Egypt	AML (+)	[71], [70]
6	Cyp3A5	Egypt	AML (+)	[72]
7	DNMT3A	Iraq	AML (+)	[73]
8	DNMT3B	Morocco	AML (+)	[74]
9	ERCC2	Iraq	AML (+)	[75]
10	Factor V Leiden	Egypt	AML (+)	[28]
11	GSTM1	Sudan	AML (+)	[76]
12	GSTT1	Sudan	AML (+)	[76]
13	IL-10	Egypt	AML (+)	[77]
14	IL-17A	Egypt	AML (+)	[78]
15	IL-17F	Egypt	AML (+)	[78]
16	IL-4	Egypt	AML (+)	[79]
17	IL 23	Egypt	AML (+)	[80]
18	JAK2	Saudi Arabia	AML (+)	[81]
19	MDM2	Egypt	AML (+)	[82], [83]
20	MDR1	Iraq	AML (+)	[45]
21	MTRR	Saudi Arabia	AML (+)	[84]
22	NAT2	Egypt	AML (+)	[85]
23	NQO1	Egypt	AML (+)	[86]
24	P21	Egypt	AML (+)	[82], [83]
25	RAD51	Egypt	AML (+)	[87]
26	Ras mutations	Iraq	AML (+)	[53]
27	SDF-1	Egypt	AML (+)	[88]
28	TCF3-PBX1	Iraq	AML (+)	[53]
29	TET2	Egypt	AML (+)	[89]
30	VDR FOKI	Egypt	AML (+)	[90]
31	XPD	Egypt	AML (+)	[91]
32	XRCC1	Egypt	AML (+)	[92], [93]
33	XRCC3	Egypt	AML (+)	[87], [94]

Among these 33 genes, adults with the JAK2 (V617F) rs77375493 F allele showed an 88-fold increase in risk for developing AML, while those with the VF genotype showed a 19-fold increase [81] (Appendix B). Other genes variants showing strong association to AML were CYP3A4 (A290G) [71], CYP2B6 + CYP3A4 [70], MDM2 T309G + P21 ser31arg [83], XRCC1 [92], VDR FOKI) [90], and NQO1 (C609T) [86]. A variant of CYP1A1 was associated with reduced risk for AML [69].

Genes Associated With CLL Among the Arab Population

For CLL, 11 studies examined the genetic association with CLL in the Arab population (Table 1). All these 11 studies were case-control studies except for one. All the case-control studies were carried out on adult CLL patients (>18 years), and the cross-sectional study examined children CLL patients (<18 years). From these studies, variants of seven genes were found to be associated with increased risk for CLL (Table 4). Four studies showed no significant genetic association with CLL and hence were not included in Table 4 [95-98].

**Table 4:** Genes significantly associated with CLL in Arab countries. CLL, chronic lymphocytic leukemia

Sr. no.	Gene	Country	Type of association with CLL (+/-)	Reference
1	ACE	Sudan	CLL (+)	[99]
2	CD19	Iraq	CLL (+)	[25]
3	CD38	Egypt	CLL (+)	[100]
4	IL-10	Egypt	CLL (+)	[101]
5	miR-196a2	Egypt	CLL (+)	[102]
6	TP53	Sudan	CLL (+)	[103]
7	VDR FokI	Sudan	CLL (+)	[104]

One study was found per gene thus indicating a need for more studies to determine the genetic landscape of CLL in this ethnic group. On review, it was found that miR-196a2 (rs11614913) with the CC genotype had the strongest association in developing CLL. Adults with the CC genotype had a ~9 times higher risk of developing CLL [102]. Additionally, individuals with CD38 (rs1800561 CG+GG genotype) exhibited a ~ 6-fold increase in the risk of developing CLL [100]. IL-10 (rs1800896 GG genotype) appeared to increase the chance of developing CLL by ~ 8-fold [101] (Appendix C).

Genes Associated With CML Among the Arab Population

In this review, 24 studies investigated the genetic links to CML among the Middle Eastern population (Table 1). Of these, 22 studies were case-control studies, one was a cross-sectional study and one was a retrospective observational study. Moreover, among these studies, only two case-control studies included children in their sample for identifying genetic association with CML.

On reviewing these articles, 14 genes were found to be associated with CML. Variants of these genes were associated with increased risk for CML except for two genes viz. CYP2D6 and NAT2 (Table 5). Additionally, a discrepancy in the association of GSTT1 was noted as two studies showed it increased the risk for CML while two other studies indicated the gene variants as protective against CML (Appendix D). Additional studies would be needed to determine the relation between this gene and CML. Three studies showed the variants of genes TET2, MTHFR, and GSTP1 were not associated with CML and hence were not included in Table 5 [126-128].

**Table 5:** Genes significantly associated with CML in Arab countries. CML, chronic myeloid leukemia

Sr. no.	Gene	Country	Type of association with CML (+/-)	Reference
1	CYP1A1	Sudan, Syria	CML (+)	[105], [106]
2	CYP2D6	Sudan	CML (-)	[105]
	CYP2D6*4	Egypt	CML (+)	[107]
3	CYP3A5	Sudan	CML (+)	[108]
4	GSTT1	Sudan, Syria	CML (+)	[109], [110], [111], [112]
	GSTT1	Iraq	CML (-)	[113], [114],
5	GSTM1	Sudan, Syria	CML (+)	[109], [111], [106], [112]
6	GSTP1	Iraq, Egypt	CML (+)	[115], [116]
7	VDR	Sudan	CML (+)	[117]
8	Caspase9	Iraq	CML (+)	[118]
9	CASP8	Iraq	CML (+)	[118]
10	MTHFR	Sudan, Egypt, Jordan	CML (+)	[119], [120], [121], [122]
11	KIT	Tunisia	CML (+)	[123]
12	NAT2	Sudan	CML (-)	[124]
13	MDR1	Morocco	CML (+)	[125]
14	XPD	Sudan	CML (+)	[109]

Four studies each were identified for GSTT1, GSTM1, and MTHFR. The gene CYP1A1 exhibited the strongest association with CML (Appendix D). Individuals carrying the CYP1A1 AG (Ile/Val) genotype or the G/G, Val/Val genotype demonstrated a 23-fold and 18-fold increased susceptibility to developing CML, respectively [105]. CYP3A5*3/*3 genotype showed a ~ 12-fold increase in risk for developing CML [108]. Additionally, MTHFR (C677T), a gene associated with the regulation of one-carbon metabolism and DNA synthesis and repair, also exhibited an increase in risk [119].

Discussion

This scoping review identified several genetic polymorphisms linked to an increased risk of leukemia in Middle Eastern countries. This review can be a useful resource for researchers who wish to replicate and compare data obtained through different methods to identify, confirm, or refute the genetic associations described here. Genetic risk is one of the major factors that contribute to the risk of leukemia alongside smoking, exposure to specific chemicals, prior history of chemotherapy, radiation exposure, rare congenital conditions, certain blood disorders, age, and gender [5,129]. Numerous genes have been identified to be linked with leukemia, and this association varies based on the specific subtype of leukemia. The complexity of leukemia as a group of hematological malignancies is reflected in the diverse genetic alterations that contribute to its initiation and progression. The identification of these associated genes is crucial for a comprehensive understanding of the underlying molecular mechanisms involved in the development and progression of leukemia. It also reveals targets for therapeutic interventions and the development of personalized treatment strategies based on the specific genetic characteristics of individual cases. Further understanding this variation as per the ethnic group would help in advancements in precision medicine, aiming to tailor treatment approaches to the unique genetic makeup of leukemia patients.

The incidence and mortality rates of leukemia vary as per ethnicity [6] indicating a probable significant role played by the genetic variations associated with the disease [4,5]. This review gives an assessment of the studies examining genes predominately increasing the risk for leukemia individuals in Middle Eastern Countries. Most of the data extracted in this review were from studies carried out in Egypt, Iraq, and Sudan. Thus acute leukemia was found to be the most commonly studied leukemia.

This review indicated that the ARID5B gene has a strong association with ALL. ARID5B is also known as MRF2 (modulator recognition factor 2) or DESRT and is part of the AT-rich interactive domain (ARID) protein family, which functions as epigenetic regulators by binding to specific or nonspecific AT-rich sequences within the genome. It also plays a critical role in cell growth and differentiation of B-lymphocyte progenitors thus further emphasizing its vital role in the development of ALL [130,131]. Therefore, highlighting the need for further studies to investigate its underlying mechanisms in the disease.

For AML, JAK2 (V617F) was found to be significantly associated with the onset of the disease. This gene plays a role in cellular growth and proliferation, thus reinforcing its vital role in the development of AML [132]. Numerous studies have confirmed a strong association of JAK-STAT mutation with hematologic disorders and leukemia [133] thus, further reinforcing the findings of this review. Additionally, one more gene MDM2 was found to be strongly linked with the risk for AML [83]. This gene is an oncoprotein-blocking tumor suppressor protein p53 [134]. MDM2 via its activity is connected to the JAK-STAT pathways. Targeted treatment to MDM2 has been shown to rapidly reduce JAK2 V617F allele burden [135]. Among the genes reducing the risk for AML, ERCC2, which is part of the DNA repair and nucleotide excision repair (NER) pathway [136], was also found in the analysis of the present in the Arab population. By further investigating the strongly associated and protective genes, a better understanding of AML would be ensured. Thus, warranting a better prognosis of AML in the Arab population.

This study also showed miR-196a2 to be strongly associated with increasing the risk of CLL in the Arab population. This gene plays a vital role in the developmental signaling pathway making it a crucial target to be further investigated for its relation with CLL [137]. The analysis further revealed that CD38 and IL-10 genes also have a strong association with CLL among the Arab population. Similar analysis revealed CYP1A1 and MTHFR to be associated with an increased risk of CML among the Arab population. MTHFR is associated with the regulation of one-carbon metabolism and DNA synthesis and repair, whereas CYP1A1 is involved in metabolism. Genetic variations in CYP1A1 modify enzyme function, impacting its ability to metabolize carcinogenic and mutagenic chemicals and in turn affecting one’s susceptibility to developing leukemia [138].

It should be noted that the studies included in this review were predominately carried out in Egypt (56 out of 119). As a result of the research studies lacking in other regions, there is a possibility that the reviewed studies may underrepresent genetic associations with various types of leukemia among racial and ethnic minorities, who have been underrepresented in research. This finding also highlights the need for a comprehensive study covering all the countries of the Middle East to get an accurate representation of the genetic associations of leukemia.

The limitation of this review was that the search was confined to only two databases viz. PubMed and Google Scholar. However, these databases are known to be comprehensive and the chances of missing relevant articles would be almost negligible. Additionally, this review comprehensively covers the major types of leukemia and provides a solid foundation for researchers aiming to understand the genetic variations associated with the disease in the Arab population. Another limitation of the study is the selection of the Arab population. The studies selected were carried out in the Arab countries however there is a possibility of other nationalities/ethnicities to be included as a result of migration and treatment-seeking behavior from neighboring countries. Additionally, the ethnic composition of the Arabs is diverse as an account of the historical events [139]. Authenticating the population's ethnicity in the selected studies is beyond the scope of this study. Thus, this once again highlights the need for an extensive study to confirm the ethnicity and subsequent genetic predisposition to leukemia in the Arab region.​​​​​

## Conclusions

In summary, this review provides an overview of research published about genetic association with leukemia and Middle Eastern countries. Several genetic variants are associated with an increased risk of leukemia. However, the review also highlights the regional disparity in the research carried out. Thus, using this study as the basis, further larger-scale studies are recommended to establish the genetic associations of leukemia by utilizing a sample representative of the 22 Arab countries. The findings from this study will also work as a foundation for large-scale studies related to targeted and personalized treatment to improve the outcomes and quality of life for individuals affected by leukemia in this region. Further exploring the environmental factors interacting with these genetic variations could provide a comprehensive understanding of leukemia etiology in Middle Eastern populations. Additionally, implementing targeted screening programs based on these genetic markers might facilitate early detection and personalized treatment strategies. Ultimately, elucidating the intricate interplay between genetics, environment, and leukemia risk holds promise for improving healthcare outcomes in the Arab community.

## Appendices

Appendix A 

**Table 6:** Details of variations in the genes significantly associated with ALL in the Arab population.

Sr. NO.	Gene	rs ID	Genotype/ allele	Adults/children	Sample size	Country		Type of association		OR (95% CI)	P value	Reference
1.	KIR2DS4	-	AA haplotype	Children	228		Saudi Arabia	Increase in risk	-		0.0021, corrected p = 0.0042)	[44]
2.	CD19	-	TCC to ATC	Adults + children	100		Iraq	Increase in risk	-		<0.01	[25]
3.	IL-17A	rs3748067	C > T	Adults	322		Saudi Arabia	protective effect	0.53 (0.32–0.88)		0.013	[43]
4.	IL-15	rs10519612,	CC, Allele A	Adults	322		Egypt	Increase in risk Increase in risk	1.69 (1.08- 2.259) 1.51 (1.03 - 2.205)		<0.05	[42]
		rs17007695	TC CC TC + CC Allele T	Adults	322		Egypt	Increase in risk Increase in risk Increase in risk Increase in risk	1.813 (1.33 - 2.530) 2.364 (1.44 - 2.851) 3.847 (1.89 - 7.841) 2.111 (1.44 - 3.097)		< 0.001	[42]
5.	IL-10	rs1800896	AG, AG+GG vs AA,	Adults	308		Sudan	Increase in risk	3.1 (1.90 to 5.16) 4.89 (2.47 to 9.687)		<0.001	[41]
6.	HLA class I (A, B, Cw) and class II (DR, DQ) genes	-	A1-DR7, A2-B12, A9-B12 CW7-DQ1.	Adults	225		Iraq	Higher frequency	-		<0.01	[38]
	HLA-DQA1	-	0201 allele	Children	100		Iraq	Increase in risk			0.05	[39]
	HLA-DQA1	-	40101 allele					Protective			0.026	[39]
	HLA-DQB1	-	60301 allele					Increase in risk			0.05	[39]
	HLA-DQB1	-	30201 allele					Protective			0.001	[39]
7.	ARID5B	rs10821936	CT CC CT+ CC C (wild)	children	609		Egypt	Increase in risk Increase in risk Increase in risk Increase in risk	1.458(1.090 - 1.950) 2.722 (1.758 - 4.213) 1.654(1.253 2.182) 2.023(1.480 - 2.765)		<0.05	[21,22]
		rs10821936	CT CC CT+ CC C (wild)	Adults	609		Egypt	Increase in risk Increase in risk Increase in risk Increase in risk	1.601 (1.048-2.446) 2.528 (1.403- 4.555) 1.763 (1.175 -2.645) 2.104 (1.322- 3.349)		<0.05	[22]
	ARID5B	rs7073837 rs10740055 rs7089424 rs10821936 rs4506592 rs10994982 rs7896246 rs10821938 rs7923074	C/A A/C T/G T/C G/A G/A G/A C/A C/A	Children	289		Yemen	Increase in risk Increase in risk Increase in risk Increase in risk Increase in risk Increase in risk Increase in risk Increase in risk Increase in risk	1.66 (1.14–2.40) 1.60 (1.13–2.27) 1.46 (1.01–2.13) 1.69 (1.19–2.41) 1.44 (1.02–2.05) 1.58 (1.12–2.23) 1.75 (1.19–2.57) 1.48 (1.05–2.09) 1.70 (1.17–2.47)		≤ 0.05	[21]
	ARID5B	rs2893881 rs4948488	C/C A/G genotype A/G + G/G G-allele	Adults	160		Egypt	Increase in risk Increase in risk Increase in risk Increase in risk	2.732 (1.004 – 7.432) 20.891 (6.066 – 71.956) 22.085 (6.428 – 75.886) 16.868 (5.091 – 55.891)		<0.05	[23]
8.	MDR1 C3435T	-	CT +TT T	Children	94		Egypt	Increase in risk Increase in risk	2.91 (1.193-7.137 ) 3.37 (1.029-11.038)		<0.05	[33]
		-	TT vs Tt or tt	Adult	110		Iraq	Increase in risk	-		0.03	[45]
		- -	T/T C/T−T/T	Children	120		Egypt	Increase in risk Increase in risk	8.000 (1.425–44.920) 4.000 (1.367–11.703)		<0.05	[46]
		-	C/T−T/T	Adults	120		Egypt	Increase in risk	3.455 (1.195–9.990)		<0.05	[46]
		-	T allele	Children	220		Egypt	Increase in risk	1.610 (1.049-2.471)		<0.01	[47]
9.	MDR1C3435T and GSTM1 null	-	CT/TT + null	Children	94		Egypt	Increase in risk	0.672 (1.059-12.73)		0.032	[33]
10.	IKZF1	rs4132601 rs6964969 rs3731246	AG C allele GC+CC C allele	Children	289		Yemen	Increase in risk Increase in risk Increase in risk Increase in risk	2.14 (1.01–4.54) 2.50 (1.41–4.42) 2.01 (1.01–3.99) 1.55 (1.12–2.13)		<0.05	[26]
		rs4132601	T/G	Children	320		Tunisia	Increase in risk	1.54 (1.04–2.28)		0.029	[40]
11.	CDKN2A	rs3731246	G>C (GC+CC vs. GG) C allele	Children	289		Yemen	Increase in risk Increase in risk	2.01 (1.01–3.99) 1.55 (1.12–2.13)		<0.05	[26]
12.	TNFα	rs1800629	GA	Adults	330		Sudan	Increase in risk	1.84 (1.17 to 2.90)		0.009	[54]
13.	CCND1 (G870A)		AA	Children	40		Egypt	Increase in risk	15.750(1.424 – 174.25)		0.024	[24]
14.	Ras mutations	-	-	Children	66		Iraq	High frequency	-		-	[53]
15.	TCF3-PBX1	-	-	Children	66		Iraq	High frequency	-		-	[53]
16.	GSTM1	-	Null	Adults	256		Sudan	Increase in risk	3.7 (2.1086 to 6.5110)		<0.001	[32]
17.	GSTT1	-	Null	Children & adults	255		Yemen	Increase in risk	2.649 (1.589 - 4.416)		0.00	[34]
18.	GSTT1 + GSTM1	-	Present/present, Present/null, Null/Null	Adults	256		Sudan	Increase in risk Increase in risk Increase in risk	2.8 (1.68 to 4.64) 2.9 (1.61 to 5.17) 15.8 (2.86 to 86.765)		< 0.001	[32]
		-	GSTT1null /GSTM1 null	Children & adults	255		Yemen	Increase in risk	3.396 (1.832 - 6.297)		0.00	[34]
	GSTT1 + GSTM1	-	GSTT1null /GSTM1 null	Children & Adults	255		Yemen	Increase in risk	3.396 (1.832 -6.297)		0.00	[35]
19.	GSTP1 (Ile105Val)	-	(Ile/Val), (Val/Val)	Children & Adults	255		Yemen	Increase in risk	1.972 (1.194-3.259)		0.005	[35]
20.	GSTP1 + GSTM1 + GSTT1	-	GSTT1null /GSTM1 null with GSTP1(II105Val polymorphism	Children & Adults	255		Yemen	Increase in risk	4.125 (1.768 -9.626)		0.00	[35]
21.	GSTO2	-	-	Children	195		Egypt	Higher frequency in cases	-		0.017	[36]
		-	G [minor allele]	Children	195		Egypt	Increase in risk	1.521(1.006–2.298)		0.046	[36]
22.	CYP1A1*4	-	+/+ -/+,+/+	Children	386		Egypt	Increase in risk Increase in risk	6.600 (2.225 - 19.57) 2.16 (1.200 - 3.889)		0.001 0.01	[27]
23.	FOXP3	rs3761548	C/C	Children	252		Egypt	Increase in risk	3.07 (1.40–6.73)		<0.05	[29]
	FOXP3 -3279C/A	rs3761548	C/C	Children	252		Egypt	Increase in risk	3.07 (1.40–6.73)		<0.01	[30]
24.	ROR-γ	rs9017A/G	A/A, A allele	Children	252		Egypt	Increase in risk Increase in risk	3.31 (1.69–6.50) 1.56 (1.09–2.23)		<0.05	[29]
25.	GATA3	rs3824662	AA	Children	389		Egypt	Increase in risk	2.748 (1.176 - 6.424)		<0.05	[31]
26.	Factor V Leiden	-	GA GA + AA Allele A	Adults	176		Egypt	Increase in risk Increase in risk Increase in risk	2.639 (1.045–6.669) 2.828 (1.13–7.075) 2.824 (1.175–6.785)		<0.05	[28]
27	HFE H63D	-	CC CG GG	Children	70		Egypt	Increase in risk	-		0.001	[37]
28.	NQO1*2 (C609T)	-	CT CT + TT	Adults	206		Tunisia	Increase in risk Increase in risk	1.41 (1.04–1.93) 1.38 (1.05–1.83)		0.028	[51]
	NQO1 C465T	-	CT TT CT/TT	Children	235		Saudi Arabia	Increase in risk Increase in risk Increase in risk	8.41 (3.06-23.09) 6.4(1.29-31.64) 7.83 (3.27-18.75)		Not given	[52]
29.	MTHFR C677T	-	TT	adults	150		Egypt	Protective	0.48 (0.28- 0.80)		0.0051	[48]
	MTHFR C677T /MTHFR A1298C	-	CT/CC	adults	150		Egypt	Protective	0.451 (0.384- 0.529)		0.001	[48]
	MTHF1298		AC AC+CC	Children and adults	399		Egypt	Increase in risk	0.382(0.222-0.658) 0.432 (0.263-0.707)		0.001 0.002	[49]
30.	NAT2*11A C481C>T	rs1799929	CT	Children	92		Egypt	Increase in risk	1.243 (1.056–3.082)		0.034	[50]
	NAT2*7A 857G>A	rs1799931	GA GA+AA	Children	92		Egypt	Increase in risk Increase in risk	2.741 (1.301–5.776) 2.721 (1.317–5.624)		< 0.05	[50]

Appendix B 

**Table 7:** Details of the gene significantly associated with AML in Arab countries.

Sr. No.	Gene	rs ID	Genotype/ allele	Adults/children	Sample size	Country	Type of association	OR (95% CI)	P value	Reference
1	CYP1A1	-	GG	Adults	265	Sudan	Protective	0.024 (0.001 - 0.737)	0.033	[69]
2	CYP3A4 (A-290G)	-	AG AG+GG	Adults	149	Egypt	Increase in risk Increase in risk	11.429 (2.513–51.974) 18.857 (4.041–78.903)	<0.002	[71]
	CYP3A4	-	AA vs AG + GG	Adults	100	Egypt	Increase in risk	3.8 (1.40-10.10)	0.006	[70]
	CYP2B6 + CYP3A4	-	Combined vs single	Adults	100	Egypt	Increase in risk	14.8 (1.8-124.2)	0.003	[70]
3	Cyp3A5*3	-	heterogenous Variant	Adults	100	Egypt	Increase in risk	4.72 (1.48-15.03)	≤ 0.05	[72]
			homo Variant	Adults	100	Egypt	Increase in risk	5.23 (1.81 - 15.14)	≤ 0.05	[72]
4	CYP2B6 (G516T)	-	GG vs GT + TT	Adults	100	Egypt	Increase in risk	3.0 (1.3-6.9)	0.008	[70]
5	SDF-1 G801A	rs1801157	AG, AA A	Adults	120	Egypt	Increase in risk Increase in risk	3.5 (1.555-7.874) 3.134 (1.521-6.459)	0.002 0.002	[88]
6	SDF-1 + CXCR4	-	CXCR4positive and SDF-1 AG+AA	Adults	120	Egypt	Increase in risk	4.274 (1.161-15.738)	0.031	[88]
7	DNMT3A	rs11695471	AT or AA vs TT(wildtype)	Adults	80	Iraq	Increase in risk	-	0.02	[73]
8	DNMT3B	rs 1569686	GT/TT	Adults	321	Morocco	Increase in risk	1.72 (1.012.95)	0.04	[74]
9	MDR1 (C3435T)		TT vs Tt or tt	Adult	110	Iraq	Increase in risk	-	0.03	[45]
10	Factor V Leiden	-	GA GA + AA Allele A	Adults	176	Egypt	Increase in risk Increase in risk Increase in risk	2.639 (1.045–6.669) 2.828 (1.13–7.075) 2.824 (1.175–6.785)	<0.05	[28]
11	MDM2 SNP309	-	TG+GG	Children & Adults	149	Egypt	Increase in risk	-	0.07	[82]
	MDM2 T309G + P21 ser31arg	-	both mutant	Children & adults	149	Egypt	Increase in risk	6.807 (1.909-24.629)	0.003	[83]
12	P21 codon 31	-	p21 arg/arg, p21 ser/arg, p21 ser/ser P21 (ser/arg+arg/arg)	Children & Adults	149	Egypt	Increase in risk	-	<0.05	[82]
	P21	-	ser/arg ser/arg+arg/arg	Children & adults	149	Egypt	Increase in risk Increase in risk	2.946(1.216-7.134) 3.07 (1.32-7.138)	<0.05	[83]
13	MTRR (A66G)	-	AG GG AA vs AG + GG G allele	Adults	200	Saudi Arabia	Increase in risk Increase in risk Increase in risk Increase in risk	4.08 (2.01–8.29) 4.59 (1.87–11.24) 3.43 (1.45–8.11) 3.41 (2.11–5.5)	<0.01	[84]
14	GSTM1	-	Null	not mentioned	80	Sudan	Increase in risk	2.7(1.2-6.04)	0.012	[76]
15	GSTT1	-	Null	not mentioned	80	Sudan	Increase in risk	4.93(1.6-15.07)	0.005	[76]
16	IL-10 -819T/C	rs1800871	-	Adults	165	Egypt	Higher frequency	-	<0.01	[77]
17	IL-17A G-197A	rs2275913	AA	Adults	200	Egypt	Increase in risk	2.755 (1.078 -7.042)	0.034	[78]
18	IL-17F A7488G	rs763780	AA, GA, GG	Adults	200	Egypt	Increase in risk	2.755 (1.078 -7.042)	0.034	[78]
19	IL 23		Hetero	Adults	124	Egypt	Increase in risk		0.0292	[80]
20	IL-4 intron 3 VNTR	-	P1P2 and P2P2	Adults	120	Egypt	significantly higher in AML patients	-	0.001	[79]
21	APE1Asp148Glu	rs1130409	T/G G/G T/G+G/G APE1(V) & RAD51(W)	Adults	101	Egypt	Increase in risk Increase in risk Increase in risk Increase in risk	3.463 (1.199-10.002) 3.850 (1.049–14.124) 3.568 (1.299–9.797) 4.44 (1.131–17.464)	0.022 0.042 0.011 0.03	[66]
	APE1	rs1130409	Allele G	Children & Adults	100	Iraq	Increase in risk	2.47 (1.18 – 5.19)	0.01	[67]
		rs1130409	T/T	Children & Adults	100	Iraq	Protective	0.34 (0.14 – 0.83)	0.008	[67]
22	JAK2 (V617F)	rs77375493	VF F	Adults	200	Saudi Arabia	Increase in risk Increase in risk	18.79 (2.442–144.6) 87.76 (11.76–654.7)	0.0001 <0.0001	[81]
23	NAT2 (G857A)	rs1799931	GG G allele	Adults	140	Egypt	Increase in risk Increase in risk	3.765 (1.167–12.15) 2.365 (1.344–4.163)	<0.05	[85]
24	XPD	rs13181	C/C	Children and adults	150	Egypt	Increase in risk	4.4 (1.245–15.556)	0.025	[91]
	XPD + XPC	-	-	Children and adults	150	Egypt	Increase in risk	2.333 (1.015–5.363)	0.043	[91]
	XPD + XPG + XPC	-	-	Children and adults	150	Egypt	Increase in risk	3.042 (1.179–7.849)	0.018	[91]
25	TET2	rs6843141	-	Adults	82	Egypt	Higher in patients	-	< 0.0001	[89]
26	RAD51	-	Mutant	Children & Adults	80	Egypt	Increase in risk	2.833 (1.527 - 8.983)	0.03	[87]
27	XRCC3	-	Mutant	Children & Adults	80	Egypt	Increase in risk	2.909 (1.761 - 9.788)	0.01	[87]
	XRCC3	-	-	Adults	144	Egypt	Increase in risk	2.77 (1.35 – 5.66)	0.002	[94]
28	XRCC1	rs1799782	Trp194Trp Trp	Adults	184	Egypt	Increase in risk	18.23 (1.52 - 5.27) 3.47 (1.07 -1.92)	<0.05	[92]
	XRCC1		codon 194 codon 399	Children and adults	60	Egypt	Increase in risk Increase in risk	6.15 (1.887-20.05) 4.000 (1.136-14.085)	0.002 0.025	[93]
29	CASP9 712 C > T	rs4645981	CC CT TT CT + TT	Adults	100	Egypt	Increase in risk	3.644 (1.39–9.528) 3.1 (1.2–8.0) 3.471 (1.382–8.934) 3.644 (1.39–9.528)	<0.05	[68]
30	ERCC2 (Lys751Gl)	-	Allele G Allele T	Adults	100	Iraq	Increase in risk Protective	2.15( 1.05–4.43) 0.46 (0.23–0.96)	0.03 0.03	[75]
31	VDR FOKI	-	Ff f allele	Adults	60	Egypt	Increase in risk	5(1.4-16.8) 4.71 (1.6-13.7)	<0.01	[90]
32	NQO1 (C609T)	-	CT TT CT+TT	Children & adults	182	Egypt	Increase in risk	2.4 (1.05-5.4) 5.4 (2.1-13.6) 3.4 (1.5-7.6)	<0.05	[86]
33	Ras mutations	-	-	Children	66	Iraq	High frequency	-	-	[53]
34	TCF3-PBX1	-	-	Children	66	Iraq	High frequency	-	-	[53]

Appendix C 

**Table 8:** Details of gene significantly associated with CLL in Arab countries.

Sr. No.	Gene	rs ID	Genotype/ allele	Adults/children	Sample size	Country	Type of association	OR (95% CI)	P value	Reference
1	ACE	-	I/D	Adults	80	Sudan	Increase in risk	-	0.001	[99]
2	CD38	rs6449182 rs1800561	GG CG+GG	Adults	140	Egypt	Increase in risk Increase in risk	2.813 (1.90–4.17) 5.740 (1.91–17.28)	<0.001	[100]
3	miR-196a2	rs11614913	CC, C allele	Adults	80	Egypt	Increase in risk	8.56 (2.78–26.36) 3.38 (1.75–6.56)	<0.05	[102]
4	IL-10	rs1800896	AG (hetero) GG (homo) AG + GG Allele G	Adults	100	Egypt	Increase in risk	2.148 (1.151 -4.009) 7.514 (2.99 -18.90) 2.788 (1.53- 5.09) 3.481 (1.94- 6.24)	0.016 < 0.001 0.001 < 0.001	[101]
5	TP53 Codon 72 Arg/Pro	-	Pro/Pro Pro allele frequency Arg/Pro+Pro/Pro	Adults	190	Sudan	Increase in risk	4.01 (1.57-10.26) 2.23 (1.45-3.41) 2.4 (1.3-4.43)	<0.005	[103]
6	VDR FokI	-	Ff	Adults	80	Sudan	Increase in risk	3.370 (1.070-10.613)	0.038	[104]
7	CD19	-	TCC to ATC	Adults + children	100	Iraq	Increase in risk	-	<0.01	[25]

Appendix D

**Table 9:** Details of genes significantly associated with CML in Arab countries.

Sr. No.	Gene	rs ID	Genotype/ allele	Adults/ children	No.	Country	Type of association	OR (CI)	P value	Reference
1	CYP1A1	-	G/G, Val/Val, AG (Ile/Val)	-	300	Sudan	Increase in risk	18.38 (7.4 - 45.9) 23.125 (7.2 - 73.9)	<0.05	[105]
		-	Ile/Val + Val/Val	Adults	298	Syria	Increase in risk	2.52 (1.41 + 4.5)	0.0019	[106]
			Val	Adults	298	Syria	Increase in risk	3.3 (1.96 - 5.53)	0.0001	[106]
2	CYP2D6	-	GA	-	300	Sudan	Protective	0.036 (0.01 - 0.27)	<0.05	[105]
	CYP2D6*4	-	-	Adults	90	Egypt	Increase in risk	-	0.004	[107]
	CYP1A1*2C + CYP2D6*4		AA + GA	-	300	Sudan	Protective	0.02 (0.001-0.29)	<0.05	[105]
3	CYP3A5	-	CYP3A5*3/*3	Adults	92	Sudan	Increase in risk	11.71(4.3 –31.83)	0.00	[108]
4	GSTT1	-	null	Adults	330	Sudan	Increase in risk	3.39 (2.0006 to 5.745)	<0 0.001	[109]
		-	Null	Children & adults	96	Iraq	Protective	0.391 (0.1741-0.9033)	0.0304	[113]
		-	Null	Not mentioned	300	Sudan	Increase in risk	2.781 (1.59 - 4.85)	0.00	[110]
			Null	Adult	219	Sudan	Increase in risk	3.25 (1.87-5.65)	0.001	[111]
			Null	Adults	298	Syria	Increase in risk	1.98 (1.12 - 3.5)	0.02	[106]
5	GSTM1	-	null	Adults	330	Sudan	Increase in risk	2.9 (1.8160 to 4.6408)	<0 0.001	[109]
			null	Adult	219	Sudan	Increase in risk	2.14 ( 1.25-3.67)	0.005	[111]
			Null	Adults	298	Syria	Increase in risk	2.55 (1.54 - 4.22)	0.00024	[106]
	GSTT1+ GSTM1+XPD	-	Null+ Null + Gln/Lys Null+ Null + Lys/Lys Present + Null + Lys/Lys	Adults	330	Sudan	Increase in risk Increase in risk Increase in risk	5.8 (2.80 to 12.08) 9.5 (1.19 to 76.04) 5.9 (1.28 to 26.89)	<0.05	[109]
	GSTM1 + GSTT1	-	Present+ Null	Adults	185	Morocco	Protective	0.3 (0.08-0.99)	0.049	[114]
	GSTM1 + GSTT1		null genotypes	Adult	219	Sudan	Increase in risk	2.57(1.44-4.56)	0.01	[111]
	GSTM1 + GSTT1	-	null genotypes	Adults	176	Syria	Increase in risk	2.12 (1.24–3.7)	0.007	[112]
			GSTM1−/GSTT1−	Adults	176	Syria	Increase in risk	3.6 (1.37–9.30)	0.01	[112]
			GSTM1+/GSTT1−	Adults	176	Syria	Increase in risk	2.9 (1.45–5.6)	0.0027	[112]
			GSTM1-/GSTT1-	Adults	298	Syria	Increase in risk	5.47 (2.18 - 13.68)	0.0001	[106]
			GSTM1-/GSTT1+	Adults	298	Syria	Increase in risk	1.97 (1.1 + 3.52)	0.025	[106]
6	GSTP1 (Ile105Val)	-	Variant	Adults	80	Iraq	Increase in risk	3.095(1.24 – 7.7)	0.014	[115]
	GSTP1	-	mutant allele	Adults	70	Egypt	Increase in risk	3.889(1.417–10.674)	0.009	[116]
7	VDR	-	Fok-I f/f	Adults + children	77	Sudan	Higher frequency	-	0.000	[117]
8	Caspase9 (293del)	rs4645982	222(-/-) (homozygous of deletion)	Adults	193	Iraq	Increase in risk	3.44 (1.68_7.02)	0.001	[118]
		rs4645982	241/222 (+/-) (heterozygous of deletion)	Adults	193	Iraq	Protective	0.33 (0.16_0.72)	0.005	
9	CASP8 −652 6N ins/del	rs3834129	ins/ins (139/139)	Adults	193	Iraq	Increase in risk	3.06 (1.47-6.40)	0.004	[118]
10	MTHFR (C677TCT)		CT	Adults	300	Sudan	Increase in risk	6.405 (2.437-16.84)	<0.05	[119]
	MTHFR (A1298C)		AC CC	Adults	300	Sudan	Increase in risk	2.022 (1.23 - 3.33) 17.79 (2.33 - 136.21)	<0.05	[119]
	MTHFRC677T and A1298C		CC and CC CT and AA CT and AC TT and AC	Adults	300	Sudan	Increase in risk	15.000 (1.88 - 119.62) 5.333 (1.429 - 19.901) 12.500 (2.754- 56.729) 7.667 (2.113 - 27.817)	<0.05	[119]
	MTHFR	rs 677	CT	Adults	100	Sudan	Increase in risk	-	Not given	[120]
	MTHFR C677T/A1298C	-	TT/CC	Adults	185	Egypt	Increase in risk	1.915 (1.202 -3.845)	0.02	[121]
	MTHFR C677T	-	TT	Not mentioned	319	Jordan	Increase in risk	2.84 [1.24,6.50]	0.014	[122]
11	KIT	-	2440G>A/R796K (Exon 17)	Adults	84	Tunisia	Increase in risk	-	0.004	[123]
12	NAT2 (A803G)	-	AG GG A allele	Adults	300	Sudan	Protective Protective Protective	0.044 (0.020-.095) 0.201 (0.091-.445) 0.419 (1.68-3.66)	0.00	[124]
	NAT2 (G857A)		GA AA G allele	Adults	300	Sudan	protective	0.002( 0.000-.019) 0.018 (0.002-.133) 0.13 (4.43-13.7)	0.00	[124]
13	MDR1 C1236T		CC vs TT CC/CT vs TT	Adults	188	Morocco	Increase in risk	2.7 (1–7.32) 3.3 (1.3–8.4)	<0.05	[125]
14	XPD	-	Gln/Gln	Adults	330	Sudan	Increase in risk	2.98 (1.255 to 7.0713)	0.013	[109]
